# Miscellany

**Published:** 1903-09

**Authors:** 


### Miscellany.

Hemorrhagic Diathesis controlled by Calcium Chloride.—While well aware that the above caption invites criticism, it has been selected as likely to attract attention to a case illustrating the value of this drug as a styptic published in the *British Journal of Dental Science,* vol. xlvi., May 15, 1903, page 461, and credited to the *Lancet.* Cases of hemorrhage, when this peculiar condition exists, are always dangerous; they call for prompt and reliable treatment; therefore anything which promises greater usefulness than the usual stock remedies cannot be too generally known.

The case is reported by Mr. T. Wilson Parry, M.A., M.B. (Cantab.). He was called in the early morning of September 4 to a boy aged seven years and four months, who was bleeding from the mouth. The blood was seen to be welling up from a little slit or cavity in the gum between the left lower first permanent molar and the temporary molar immediately in front of it, the latter being decayed on its distal surface. Both of these teeth were firm. There was a rapid oozing from the cleft, and evidence that there had been a continuous bleeding through the night. Without relating all the details, it is sufficient to say that the bleeding was so profuse as to bring the patient near to the danger point. During four days attempts to arrest this hemorrhage were persistently made; alum, tannic acid, turpentine, perchloride of iron, and adrenalin chloride being used without relief. Mr. Bilton Pollard, who was called in consultation, after hearing what had been done, suggested a trial of calcium chloride. A small pledget of cotton was steeped in a solution of this (thirty grains to the ounce) and inserted in the little cavity in the gum from which the blood came. This was shortly replaced by another, and yet again by several others, until the removed plug showed no blood-stain whatever. A final plug was, of course, left *in situ.* The cessation of bleeding was now complete. The nurse in attendance was then instructed to moisten the inserted plug with some fresh solution every twenty minutes. This was continued from eight to ten o'clock P.M., when the boy fell asleep and did not awake until six o'clock the following morning, there having been in the mean time no return whatever of the hemorrhage, notwithstanding that the plug was gone; nor did it again occur.

The family history given leaves no doubt but that it was a case of hæmophilia.

While the use of calcium chloride as a styptic is not unknown, it is safe to say that it is not generally appreciated, nor yet is it generally "put down in the books" as a valuable agent in such cases as this. Not only is it efficient and cleanly, but being non-toxic, or nearly so, gives it additional value when a styptic is called for to arrest hemorrhage in the mouth of a child. Should a portion be swallowed, it is not at all likely that the amount would exceed a reasonable dose of the drug, and, so far as being injurious, it is the very drug one would prescribe.

In concluding, he says, "It is into the hands of general practitioners that trying and anxious cases as the one recorded first fall, and it is for such as these who may be, as I myself was, unaware of the unquestionable superiority, at least in this instance, of calcium chloride as a local styptic over other styptics of more familiar name and repute, that I take the liberty of publishing this case."—W. H. T.

---

Celluloid Splint for supporting Loose Teeth.—At a recent meeting of the New York Odontological Society Dr. M. L. Rhein demonstrated a method of using celluloid for this purpose. He first binds the teeth together with ligatures of pure silver wire about 30 gauge. To prevent this being displaced, he cements little buttons of zinc phosphate cement upon the necks of the teeth. This he covers with celluloid dissolved in acetone, which not only hides the silver wire from view, but also holds the teeth much more secure, makes the ligatures far more cleanly, and, as it hardens with a smooth surface, is much more comfortable to the patient. Acetone is a perfect solvent for celluloid, and as a solution for this purpose can be quickly made, the doctor prefers to make it fresh for each case. He directs that as much celluloid be dissolved in the acetone as it will take up. To give the solution more body, and to give it a more natural tooth-color, he adds tin oxide. It may be made of a thicker or thinner consistency to suit the case or the operator's preference. It requires a very long time to set, and should be kept dry about an hour. By that time it assumes a cartilaginous condition, and the patient may then be dismissed, but requires more time to become quite hard. Some little experiment is needed to ascertain the proper consistency.—*Dental Cosmos,* May, 1903, page 389.

